# Adaptive platform trials in critical care

**DOI:** 10.1016/j.jointm.2024.04.002

**Published:** 2024-05-29

**Authors:** Muralie Vignarajah, Bram Rochwerg

**Affiliations:** 1 Department of Surgery, Queen's University, K7L 3N6, Kingston, ON, Canada; 2 Department of Medicine, McMaster University, L8S 4L8, Hamilton, ON, Canada; 3 Department of Health Research Methods, Evidence, and Impact, McMaster University, L8S 4L8, Hamilton, ON, Canada

## Introduction

With the development of novel therapeutic options and treatment pathways for critically ill patients, innovative methods to assess treatment efficacy in a timely manner are greatly needed. Not only that, but the traditional methods of conducting randomized controlled trials (RCTs) are often inefficient and exceedingly expensive.^[^^1^^]^ Platform trials represent an emerging trial design whereby multiple interventions are simultaneously evaluated within a single trial infrastructure. Platform trials often employ adaptive methodologies and unlike traditional RCTs, they use a Bayesian approach that relies on interim analyses and pre-set stopping rules and allows for treatment arms to be added or removed based on emerging evidence suggesting their efficacy or futility.^[^^2^^]^ Platform trials therefore are highly flexible, but at the same time can also be highly complex. The first platform trials were unveiled in the field of oncology, such as with STAMPEDE, examining the use of androgen deprivation therapy with abiraterone and prednisolone for advanced prostate cancer therapy.^[^^3^^]^ However, platform trials have now expanded into critical care, particularly since the coronavirus disease 2019 (COVID-19) pandemic. With a worldwide need for rapid therapeutic options against the virus, platform trials such as REMAP-CAP were instrumental in reducing COVID-19 mortality, as they examined the use of corticosteroids and IL-6 receptor blockers in COVID-19 critically ill patients.^[^^4^^]^ This article will discuss the benefits and potential pitfalls of using platform trials in critical care research and briefly discuss what the future of platform trials may entail.

## Benefits of Platform Trials

It has long been established that randomized controlled trials are time-consuming and expensive endeavours.^[^^1^^,^^5^^]^ One benefit of platform trials is that they enable reduced costs. By simultaneously evaluating multiple interventions within the same infrastructure, platform trials can use shared personnel, protocols, site engagement, site education, and contracts and can facilitate expedited research ethics board (REB) approvals whenever new interventions are introduced.^[^^2^^]^

Furthermore, platform trials have the ability to compare interventions against a shared control arm, in comparison to standalone traditional RCTs which require a control group for each intervention investigated. This allows for the trial to be completed with a reduced number of overall participants, compared to if the studies were done independently or consecutively,^[^^6^^]^ ultimately providing actionable results in an expedited fashion.

In addition, the adaptive nature of platform trials allows for multiple re-evaluations of interventions during the conduct of the trial. These re-evaluations occur at specific pre-set intervals, based on simulations and Bayesian analysis, and enable interventions to be removed based on pre-defined stopping rules. Therefore, rather than waiting for the entire trial to be completed, if an interim analysis suggests that an intervention has a high likelihood of being futile or harmful, it can be excluded from the adaptive trial, and a new more promising intervention is added. This enables improved trial efficiency, with the investigator able to focus on the most promising intervention arms, and potentially improves trial safety, with participants not being subjected to more harmful interventions.

Lastly, platform trials, with their increased flexibility and novel opportunities for funding, may enable improved international collaborations and partnerships.^[^^7^^,^^8^^]^ Of course, despite their many benefits, platform trials have several challenges around implementation and interpretation that can lead to difficulties for both researchers and end-users.

## Challenges of Platform Trials

The first major challenge associated with adaptive platform trials is their complexity. Patients, substitute decision-makers, and research ethics boards may have difficulty understanding the structure of platform trials, which may ultimately result in lengthy and laborious consent discussions. Similarly, clinicians themselves may struggle to comprehend the trial design and so may find it challenging to apply the trial results to their daily practice.

Second, platform trials are likely best suited for single disease states, as seen with GBM AGILE,^[^^9^^]^ focused on glioblastoma multiforme therapeutics, or Healey ALS, focused on amyotrophic lateral sclerosis management.^[^^10^^]^ However, platform trials can be harder to apply to syndromes or heterogenous populations, which are more common in critical care settings (i.e. sepsis).

Third, governance, leadership and recognition can be cumbersome in adaptive trials, especially in large multinational platforms. Since platform trials may involve numerous industry partners, the authorship, ownership, and presentation timelines of the data obtained in a platform trial need to be clearly defined up front, sometimes with the added burden of complex contracts. Moreover, many funders have yet to develop a comfort level with these trial designs so finding funding sources to establish platform trials can prove to be challenging.

Fourth, platform trials utilize complex Bayesian mathematics, to determine intervention efficacy and futility criteria, which necessitate the use of statisticians for trial setup and data interpretation.^[^^11^^]^

Lastly, platform trials also tend to run for longer periods of time, with a median trial duration of 59 months,^[^^12^^]^ The RECOVERY trial,^[^^13^^]^ for example, which examined COVID-19 therapeutics, ran for several years. As a result of this increased trial length, there is potential for loss of trial enthusiasm, site burnout, and increased turnover of local primary investigators.

## Conclusions

Despite their complexity, long duration, and complex funding, platform trials have demonstrated utility in answering clinical questions more efficiently and cost-effectively. New international critical care platform trials are being established in several areas of critical care, including sepsis and ARDS.^[^^14^^]^ The authors are currently involved with the CORT-E2 trial examining the role of corticosteroids in non-COVID-related acute hypoxemic respiratory failure, performed as part of the PRACTICAL platform trial (www.practicalplatform.org).^[^^15^^]^ PRACTICAL also has domains focused on optimizing invasive mechanical ventilation, and exploring the optimal use of extracorporeal life support.

Understanding of platform trial design remains a significant barrier to adoption. However, platform trials have the potential to improve the care we provide to critically ill patients and transform the way we perform research for years to come.

## Author Contributions

**Muralie Vignarajah:** Writing – review & editing, Writing – original draft. **Bram Rochwerg:** Writing – review & editing, Writing – original draft.
